# Choroidal neovascularization emerged right from the focal choroidal excavation in eyes with central serous chorioretinopathy post half-dose photodynamic therapy: a case report

**DOI:** 10.1186/s12886-019-1081-6

**Published:** 2019-03-08

**Authors:** Yang Liu, Xin Wang, Min Zhu, Gezhi Xu, Lei Li

**Affiliations:** 1 Department of Ophthalmology, Eye and ENT Hospital of Fudan University, Shanghai, 200032 China; 2 Shanghai Key Laboratory of Visual Impairment and Restoration, Eye and ENT Hospital, Shanghai Medical College, Fudan University, Shanghai, 200032 China

**Keywords:** Central serous chorioretinopathy, Focal choroidal excavation, Choroidal neovascularization, Photodynamic therapy, Safety profile, Case report

## Abstract

**Background:**

Focal choroidal excavation (FCE) is a common concurrent disease with central serous chorioretinopathy (CSC) and choroidal neovascularization (CNV). Photodynamic therapy (PDT) was able to cease the course of CSC with efficacy and safety. To retrospectively observed and followed up a special course in eyes with CSC and concurrent FCE treated by a half-dose of PDT.

**Case presentation:**

In this case report analysis, two eyes with CSC and concurrent FCE treated with half-dose PDT, were followed up with monthly retinal fundus examinations. Best corrected visual acuity (BCVA) and ophthalmic fundus examination were examined, including fundus photos, optical coherence tomography (OCT) and angiography. In Case 1, a 46-year-old female has been diagnosed as CSC and concurrent FCE. The baseline BCVA was 10/20. After a half-dose of PDT, complete resolution of SRF was achieved at one-month with stable BCVA. At 3 months, the patient complained of obvious metamorphosis. Multimodal images confirmed the existence of CNV, derived from the FCE, inside the zone of PDT irradiation. The development of CNV stopped promptly 1 month post the injection of ranibizumab. In Case 2, a 39 year-old male was diagnosed as bilateral CSC. The BCVA was 8/20 (od), and 16/20 (os). The multimodal images showed classic CSC manifestation in left eye, but atypical manifestation in right eye with subtle SRF and FCE. Post half-dose treatment, the SRF in left eye completely resolved at three-months, and the BCVA improved to 24/20. However, a lesion of CNV grew in the FCE after 1 month in right eye, with decreased BCVA, 4/20. One month post-injection of ranibizumab, obvious regression was witnessed, with improved BCVA, 6/20. The CNV proceeded to be a scar 2 months after injection. The BCVA maintained at 8/20.

**Conclusions:**

In this study, type II CNV was induced in two cases of CSC concurrent with FCE in 3 months post half-dose PDT. The CNV grew right from the FCE, inside the zone of PDT irradiation.

## Background

Since a new entity – focal choroidal excavation (FCE) – was discovered by Jampol in 2006 [1], its nature has been a mystery. Some authors once thought FCE changed little over time [2, 3], while more recently others reported that FCE accompanied with or progressed to chorioretinopathy over an observation period [4–6]. Several studies reported the prevalence of FCE in central serous chorioretinopathy (CSC) and found that CSC with FCE is not uncommon [7]. Multi-modal images disclosed aberrant choroidal circulation underlying the FCE, which was also typical in CSC [8]. Photodynamic therapy (PDT), as the main treatment for CSC, seemed to achieve favourable effects in eyes with CSC and FCE [5]. Choroidal neovascularization (CNV) also was a common concurrent disease with FCE. Several studies reported CNV grew in eyes with FCE during observation [4]. In this article, we reported two cases of eyes with CSC and concurrent FCE that also developed CNV shortly after half-dose of PDT. It is noted that due caution may be exercised in such cases.

## Case presentation

### Case 1

**Fig. 1:**
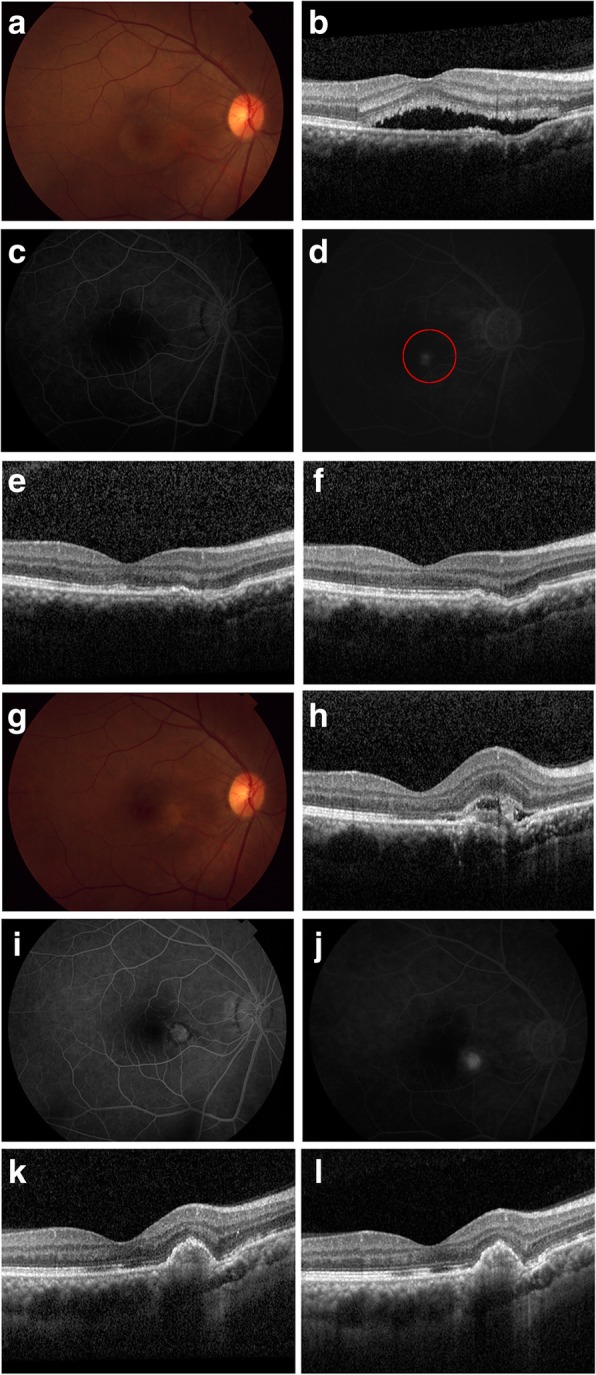
The multimodal images of case 1, a 46 year-old Chinese female with CSC and FCE. **a**. at baseline, the fundus showed serous detachment and depigmentation zone nasal to it at the macular. **b**. The OCT presented with SRF and FCE. The region temporal to FCE presented with flat irregular pigment epithelial detachment (PED). The choroidal thickness (CT) on fovea and underlying FCE was 338 μm and 202 μm. **c**, **d**. The FA from early phase to the middle showed classic inkblot leakage. The PDT irradiation was labeled in a red circle, with a diameter of 2500 μm. **e**. One month post-PDT, the OCT presented with complete resolution of SRF and RPE decompensation underlying FCE. The CT on fovea and underlying FCE was 315 μm and 183 μm. **f**. At 2 months, the reflection of RPE on OCT became clear. The CT on fovea and underlying FCE was 285 μm and 168 μm. **g**. At 3 months, the fundus presented with macular edema and still existed hypopigmentation zone. **h**. The OCT presented with a new lesion of CNV through RPE/Bruch complex and sub-retinal fluid. The CT on fovea and underlying FCE was 289 μm and 175 μm. **i**. The early phase FA showed “lacy” hypofluorescence around slightly- hyperfluorescence areas. **j**. The middle phase FA showed the obvious but focal fluorescence leaking in CNV lesion. **k**. One month post intravitreal injection, the OCT presented with RPE elevation with hyperreflective material in once CNV lesion, and only a little residue FCE nasal to the scar. The CT on fovea and underlying FCE was 282 μm and 172 μm. **l**. Two months post injection, the OCT images presented little changes, compared with last visit. The CT on fovea and underlying FCE was 280 μm and 171 μm. The best corrected visual acuity (BCVA) was 10/20 throughout the whole follow-up

A 46-year-old Chinese female patient presented with 5 months of central scotoma in her right eye, without prodromic symptoms. There was no history of traumatic, systematic illnesses, or a family history of eye disorders. Visual acuity of the afflicted eye was corrected from 8/20 to 10/20. The anterior segment and vitreous were normal. The fundus photo (Topcon TRC50LX; Topcon,Tokyo, Japan) results showed atypical pigmentary alterations and local serous detachment involving the central fovea (Fig. 1). The optical coherence tomography (OCT, Heidelberg Engineering, Heidelberg, Germany) revealed persistent sub-retinal fluid (SRF) and FCE (Fig. 1).

This patient had been diagnosed with CSC 2 months before and had been given the order for observation, but no visual benefits occurred until this visit. The fluorescent angiograph (FA) displayed the typical inkblot leakage of classic CSC. Given the symptoms duration, which had lasted for more than 3 months, half-dose PDT was chosen to halt the development of the disease. After obtaining written informed consent, a half-dose (3 mg/m^2^) of PDT (Opal Photoactivator; Lumenis, Beijing, China) was performed as a standard protocol [9], with a spot size of 2500 μm covering the leaking sites juxta-fovea, which involved the FCE.

One month post-treatment, the best-corrected visual acuity (BCVA) was 10/20. The OCT presented a complete resolution of the SRF. However, after 3 months, the patient complained of obvious metamorphosis, though the BCVA result was stable. The fundus photo showed sub-macular haemorrhage and a round hypopigmentary site inferior-nasal to the fovea. The OCT revealed, right in the FCE, a lesion of CNV that had broken through the retinal pigment epithelium (RPE), accompanied by SRF, haemorrhage and limited retinal edema. The FA confirmed the occurrence of CNV. Promptly, the patient received an intravitreal injection of anti- vascular epithelial growth factor (anti-VEGF) – ranibizumab for 0.5 mg. One month post-injection, the CNV had become a scar precisely at the sites of the FCE, and presented as an RPE elevation with hyper-reflective material. Only at the area nasal to the scar was there a little residue FCE. Neither the BCVA nor metamorphosis changed. No obvious changes occurred 3 months after injection.

### Case 2

**Fig. 2:**
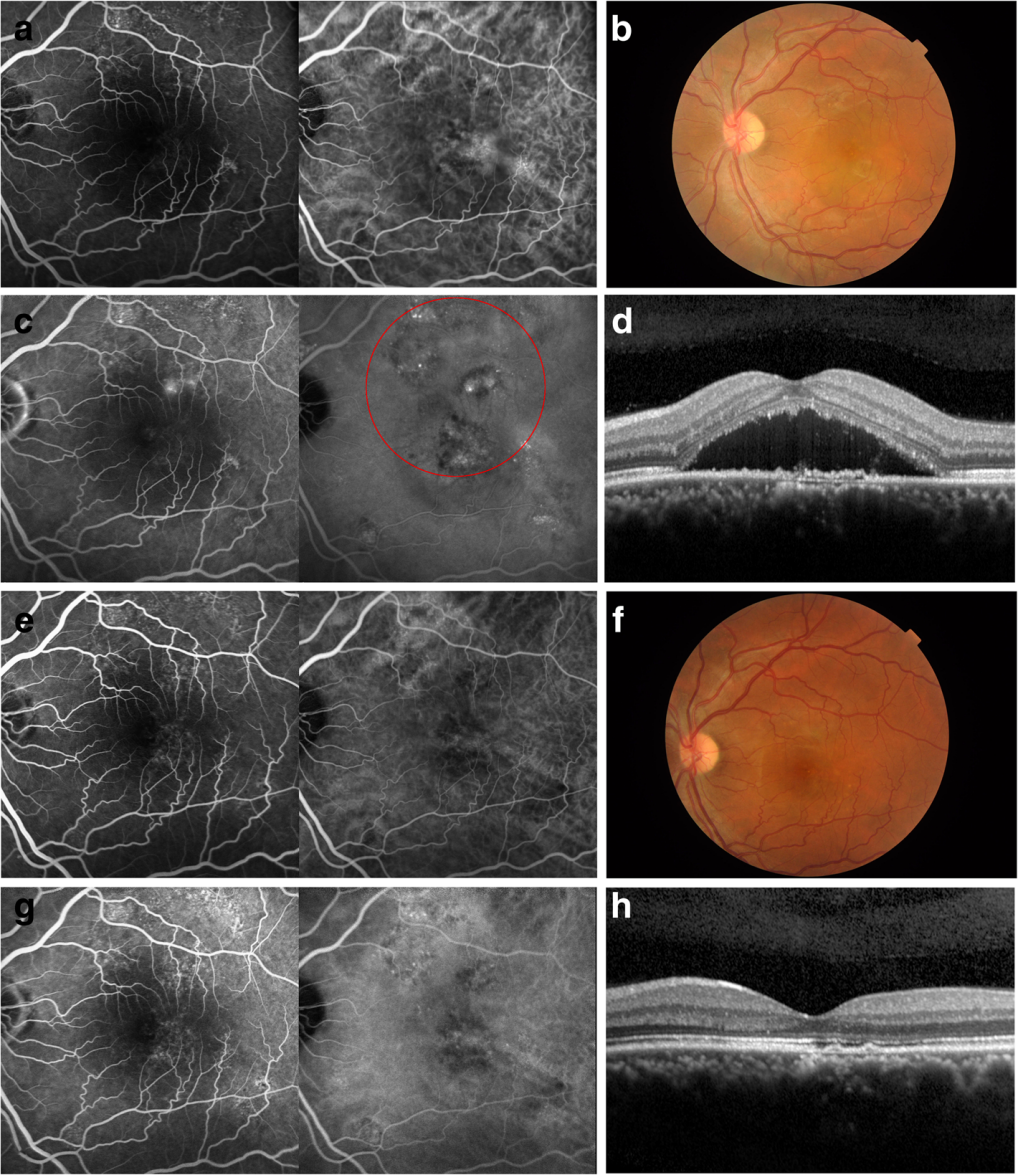
The multimodal images of left eye in case 2, a 38 year-old Chinese male patient with CSC. **a**. The early phase angiography showed widespread hyperfluorescence in FA and choriocapillary dilation in ICGA. **b**. The fundus photo displayed with tortured vessels and oval serous retinal detachment at the fovea. **c**. In middle phase, it presented with fluorescence leaking in FA and hyperfluorescence in corresponding zone in ICGA. The PDT irradiation was labeled in a red circle in ICGA, with a diameter of 5400 μm. **d**. The OCT showed SRF and limited RPE decompensation. The best corrected visual acuity (BCVA) was 16/20. **e**, 6 months post-treatment, the early phase angiography showed window defeats in FA, and choriocapillary dilation in ICGA. **f**. The fundus photo showed the disappearance of SRF. **g**. the mid-phase angiography showed no active leakage. h. The OCT presented with the complete resolution of SRF. The BCVA improved to 24/20

**Fig. 3:**
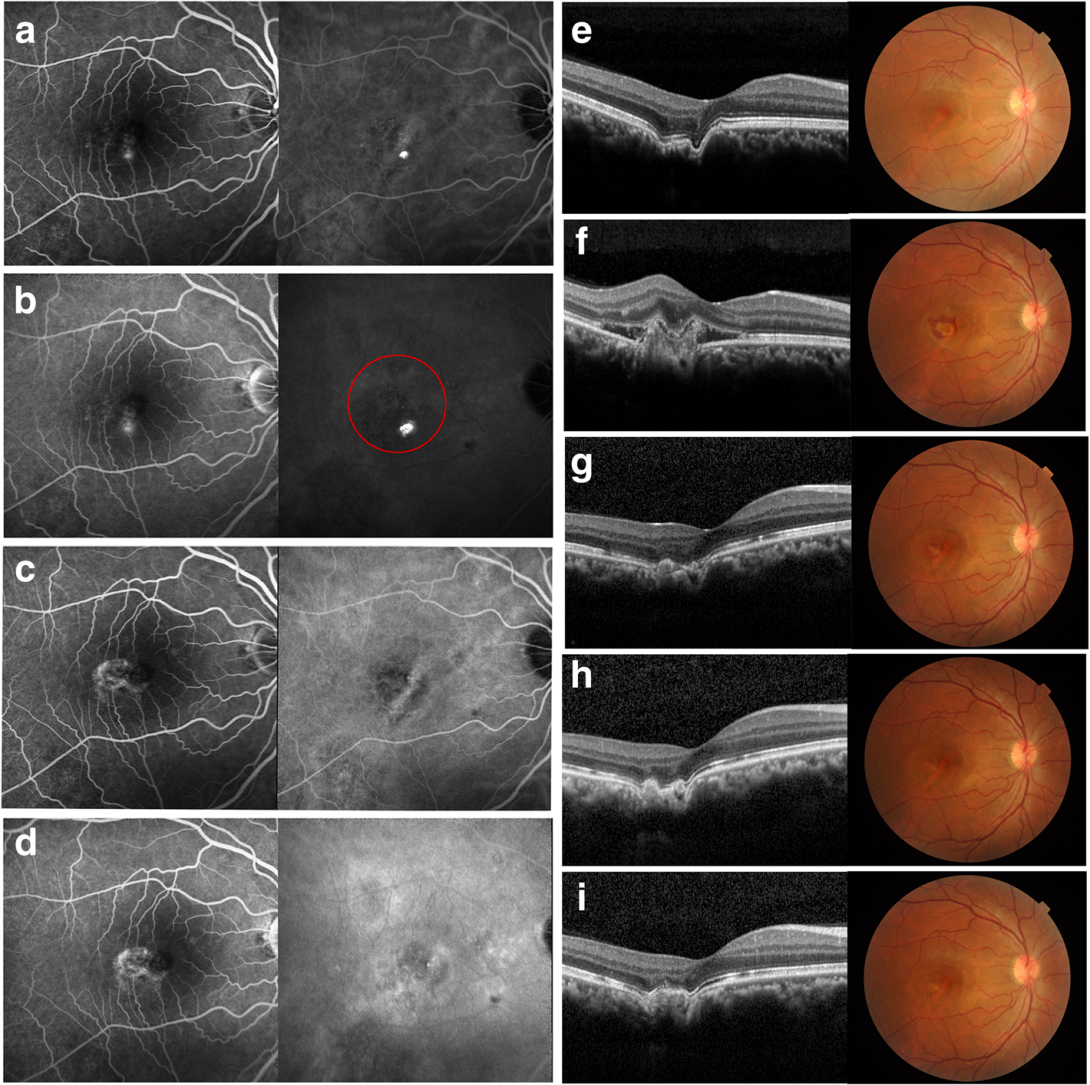
The multimodal images of case 2, a 38 year-old male with CSC and FCE in his right eye. **a**. The early phase angiography showed focal hyperfluorescence at the fovea in FA (left) and choriocapillary dilation and limited hypofluorescence in FCE in ICGA (right). **b**. In middle phase. FA (left) showed suspicous leaking on fovea. ICGA (right) showed hyperfluorescence in corresponding zone, and no obvious fluorescence abnormality was found in FCE. The PDT irradiation was labeled in a red circle, with a diameter of 3500 μm. **e**. The OCT presented with subtle SRF and FCE, corresponding to the fundus finding. The best corrected visual acuity (BCVA) was 8/20. **f**. One month post-PDT, the OCT showed CNV breaking through RPE, causing fluid accumulating sub-retina, and the fundus photo showed subretinal hemorrhage in round SRD zone. The BCVA was 4/20. **g**. One month post ranibizumab injection, the OCT showed the disappearance of CNV which corresponded to the oval zone with cross lesion in fundus finding. The BCVA was 6/20. **h**. At 2 months, the lesion developed to be a scar. The BCVA was 8/20. **c**, **d**. At 5 month post injection, the angiograph showed window defect hyperfluorescence in FA (left) and choriocapillary dilation in ICGA (right). **i**, The OCT showed two residue FCE round the CNV scar, corresponding to a cross lesion in fundus finding. The BCVA maintained at 8/20

A 39-year-old Chinese male patient presented with 6 months of vision loss in both eyes (Figs. 2, 3). There was no history of traumatic, systematic illnesses, or a family history of eye disorders. The BCVA was 8/20 (od), and 16/20 (os). The fundus findings showed pigment alteration inferior-temporal to the fovea (od) and serous retinal detachment involving the macular (os). The OCT in left eye showed SRF. The mid-phase angiography showed multi-focal leaking spots in the FA and correspondingly hyperfluorescence in the indocyanine green angiography (ICGA; Fig. 2). The OCT in the right eye showed a subtle amount of SRF with FCE. The mid-phase angiography displayed a suspicious leaking spot inferior to the fovea in the FA and correspondingly hyperfluorescence in the ICGA (Fig. 3). Given the manifestations and durations, bilateral chronic CSC was diagnosed.

After obtaining written informed consent, a half-dose of PDT was given with a spot size of 3500 μm (od) and 5400 μm (os). The spot in the right eye covered the FCE. The SRF in the left eye was partly resolved after 1 month, and completely resolved after 3 months. The angiography in the left eye presented without active leaking at 3 months. The BCVA (os) improved to 20/20 after 1 month, 24/20 after 3 months, and then remained stable at six-month.

Unexpectedly, the recovery of his right eye did not go well. After 1 month, the patient complained of further vision loss in the right eye. The fundus examination showed serous retinal detachment and subretinal haemorrhage. The OCT indicated that CNV had emerged and passed through the RPE, leading to fluid accumulation and haemorrhaging. The CNV blurred the existence of the FCE. The BCVA had declined to 4/20.The patient immediately received intravitreal an injection of 0.5 mg ranibizumab. One month post-injection, the apparent regression of the CNV and SRF was witnessed on OCT and fundus photo. The BCVA improved to 6/20. Two months later, the CNV had become a scar. The BCVA returned to baseline at 8/20. Five months after the injection, OCT and angiography confirmed the stabilization of CNV. The BCVA remained at 8/20.

## Discussion and conclusion

In this article, we observed two cases of CSC with FCE that were treated with a widely-accepted half-dose of PDT; both cases, however, instead of regressing, developed CNV through the RPE. In Case 1, the FA showed the typical inkblot fluorescence leaking, while the OCT showed SRF and concurrent FCE. One month after the PDT, the SRF had completely resolved. The regression before 2 months was indeed in accordance with the general regression of CSC after half-dose PDT. In right eye of Case 2, the OCT presented with a negligible amount of SRF and FCE at the baseline, and the golden standard angiography showed suspicious leaking in the FA and corresponding hyperfluorescence in the ICGA, seemingly in accordance with chronic CSC. Based on the multimodal results and the classic manifestation in the fellow eye, it was difficult to judge whether it was CSC with FCE, or just a non-conforming type of FCE.

Putting aside any bias in the diagnosis, the PDT, as the only intervention, appeared to be partly involved in the development of CNV. PDT with verteporfin was first used as a treatment modality for wet- age-related macular degeneration (AMD) [9]. Since the mechanism seemed to halt choriocapillary hyperperfusion, PDT was imported to reduce choroidal exudation and consequently SRF in CSC [10]. However, the standard PDT was found to enhance the expression of VEGF in choroidal epithelial cells, even leading to secondary CNV [11]. Concerning the safety profile, a modification was proposed in the treatment parameters [12, 13]. Lots of studies have confirmed the safety and efficacy profiles of CSC treated by half-dose PDT [14–16].

It is rational to cease the course of CSC with PDT because its pathogenesis was thought to be associated with aberrant choroidal circulation and hyperpermeability. As for eyes affected by CSC and FCE, it may be less wise to perform PDT, since the evidence of local choroidal ischemia has been found in some cases of FCE [8, 17]. In this study, the FCE lesion was entirely covered in the PDT irradiation. In Case 1, the sign of ischemia underlying the FCE (Fig. 1i) was obvious post treatment. In Case 2, both eyes received PDT irradiation, but the secondary CNV only grew in the eye with FCE, in spite of the larger treated area in the fellow eye. It is possible in eyes with concurrent FCE that a reduced dosage of verteporfin still induced or aggravated ischemia, though it has been proven to be safe in eyes with CSC alone [18]. Consequently, aberrant circulation may affect the overlying RPE. Molecular and anatomic changes secondary to ischemia precipitated in the growth of the CNV. The unexpected emergence of CNV necessitated switching the treatment to anti-VEGF therapy, while the well response to ranibizumab hinted that the VEGF may be involved with the development of secondary CNV.

Different outcomes were found in Luk’s study, in which visual benefits were achieved in two eyes with FCE and CSC following a half-dose of PDT [5]. To verify the safety profile in such cases, a prospective, case-controlled clinical trial with a larger sample size may be needed. Although this study is a sporadic cases report whose results are not strong enough to reach to a general conclusion for most cases, we retrospectively followed up and analysed the dynamic processes of two cases of FCE with CSC, which led to CNV and eventually to lesion scars.

In this study, we observed that type II CNV was induced in two cases of CSC concurrent with FCE in 3 months after a half-dose of PDT. The CNV grew right from the FCE inside the zone of PDT irradiation, but regressed rapidly after only one injection of ranibizumab.

## Abbreviations

AMD: age related macular degeneration
BCVA: best-corrected visual acuity
CNV: choroidal neovascularization
CSC: central serous chorioretinopathy
FA: fluorescent angiograph
FCE: focal choroidal excavation
ICGA: indocyanine green angiography
OCT: optical coherence tomography
PDT: Photodynamic therapy
SRF: sub-retinal fluid
VEGF: vascular epithelial growth factor
